# Generation of an Artificial Double Promoter for Protein Expression in *Bacillus subtilis* through a Promoter Trap System

**DOI:** 10.1371/journal.pone.0056321

**Published:** 2013-02-08

**Authors:** Mingming Yang, Weiwei Zhang, Shengyue Ji, Pinghua Cao, Yulin Chen, Xin Zhao

**Affiliations:** 1 College of Animal Science and Technology, Northwest A&F University, Yangling, Shaanxi Province, People's Republic of China; 2 Department of Animal Science, McGill University, Quebec, Canada; University of Groningen, The Netherlands

## Abstract

*Bacillus subtilis* is an attractive host for production of recombinant proteins. Promoters and expression plasmid backbones have direct impacts on the efficiency of gene expression. To screen and isolate strong promoters, a promoter trap vector pShuttleF was developed in this study. Using the vector, approximately 1000 colonies containing likely promoters from *Bacillus licheniformis* genomic DNA were obtained. Amongst them, pShuttle-09 exhibited the highest β-Gal activities in both *Escherichia coli* and *B. subtilis*. The activity of pShuttle-09 in *B. subtilis* was eight times of that of the P43 promoter, a commonly used strong promoter for *B. subtilis*. A sequence analysis showed that pShuttle-09 contained P*_luxS_* and truncated *luxS* in-frame fused with the reporter gene as well as another fragment upstream of P*_luxS_* containing a putative promoter. This putative promoter was a hybrid promoter and its β-Gal activity was higher than P*_luxS_*. Reconstructing the hybrid promoter from pShuttle-09 to P*_lapS_* further improved the β-Gal production by 60%. The usefulness of our promoter trap system is likely due to random shuffling and recombination of DNA fragments and adoption of a rapid and high-throughput screening. Thus, our data provide additional evidence to support the concept of using a promoter trap system to create new promoters.

## Introduction

*Bacillus subtilis* is a non-pathogenic gram-positive bacterium and has been an attractive host for production of recombinant proteins from both prokaryotic and eukaryotic origins [1], [2], [3]. The suitability and popularity of *B. subtilis* as one of main hosts for recombinant protein production is due to several reasons: generally recognized as safe as a result of its lack of pathogenicity and the absence of endotoxins; capable of secreting functional proteins directly into culture media, easy for genetic manipulation and handling, and capable of large-scale fermentation [2], [4], [5], [6].

*B. subtilis* own proteins can be over expressed in *B. subtilis* in the order of several grams per liter [3]. However, secretion of heterologous proteins in *B. subtilis* is usually low [7]. Thus, efficient production of high-value recombinant proteins in *B. subtilis* remains a major challenge. To circumvent the problem, strong promoters, a variety of translation/secretion signals, and transcription terminators are continuously being explored. In particular, several strong promoters have been reported [8], [9], [10], [11], [12], [13], [14], [15]. In spite of the progress, it is still beneficial to find even more potent promoters.

Promoters are important regulatory elements in a genome and control spatial and temporal expression of genes and their expression strength. In bacteria, a promoter is recognized by RNA polymerases and associated sigma factors, which may be recruited to the promoter by regulatory proteins binding to specific sites in the promoter. There are several ways to find new promoters. The advent of DNA sequencing has significantly accelerated sequencing of many bacterial genomes and prediction of promoters. However, it is not yet possible to scan a bacterial genome sequence and readily predict the expression behavior of genes belonging to a regulon, largely because an accurate prediction of promoters is difficult [16]. Alternatively, a promoter trap vector, which is a plasmid containing a multiple cloning site at the 5′ end of a promoter-less marker gene, can be used to identify promoters [17], [18], [19]. An unidentified DNA fragment is cloned into the multiple cloning site and expression of the marker gene is monitored to identify active promoter elements in the unidentified DNA fragment. Promoter trap systems have been successfully used to identify promoters for *B. subtilis* either from *B. subtilis* itself or from other bacillus species [20], [21], [22], [23].

A promoter trap system could perform shuffling and recombination of DNA fragments and screen of strong promoters simultaneously. However, this has never being reported. Fortuitously, when a promoter trap system was used in this study to screen Sau3A I cut fragments from *B. licheniformis* genomic DNA for potential promoters, a strong double promoter was identified, containing a normal promoter and a hybrid promoter due to shuffling and recombination. The putative double promoter was further improved and used for protein expression both in *E. coli* and *B. subtilis*. Our results show that promoter trap systems could be suitable for finding novel strong promoters.

## Materials and Methods

### Bacterial strains, plasmids and growth conditions

*B. subtilis* 1A747 (*B. subtilis* PY79) was a gift from Bacillus Genetic Stock Centre of the Ohio State University. *B. pumilus* BYG, *B. licheniformis* A061, *B. amyloliquefaciens* and *B. megaterium* W-5 were isolated by our laboratory. The bacterial strains were isolated from effluent of a paper mill in Hubei province of China and partial 16S rDNA sequencing was used for identification of species in the genus Bacillus. The plasmids used in this study are listed in Table 1. *E. coli* DH5α was purchased from Novagen (Darmstadt, Germany). The bacterial strains were cultured in the Luria-Bertani (LB) medium at 37°C. The following concentrations of antibiotics were used for selection: 100 µg/mL ampicillin (Amp), 5 µg/mL chloramphenicol (Cm) and 50 µg/mL spectinomycin (Spec).

**Table 1:** Plasmids used in this study.

Plasmids	Relevant characteristics	Sources
pGJ103	*E. coli-B. subtilis* shuttle vector	[6]
pDL	*bgaB* gene donor	[6]
pDG1728	specR gene donor	[27]
pShuttleF	Promoter-trapping vector	This study
pB43	P43promoter donor	This study
pShuttle-09	Recombinant cloned promoter fragment	This study
pShuttle-P43	pShuttleF inserted P43 Promoter	This study
pShuttle-luxi	*bgaB* directed by PluxS of *B. licheniformis* in shuttle vector	This study
pShuttle-luxS	*bgaB* directed by PluxS of *B. subtilis* in shuttle vector	This study
pShuttle-luxP	*bgaB* directed by PluxS of *B. pumilus* in shuttle vector	This study
pShuttle-luxM	*bgaB* directed by PluxS of *B. megaterium* in shuttle vector	This study
pShuttle-Hyb	*bgaB* directed by hybrid promoter from pShuttle-09	This study
pShuttle-Fus1	pShuttleF harbouring cloned promoter without of 5′end of *luxs* CDS	This study
pShuttle-PLapS	pShuttle-F harbouring combined promoter P*_LapS_*	This study
pShuttle-PLapP	pShuttle-F harbouring combined promoter P*_LapP_*	This study
pShuttle-PLapL	pShuttle-F harbouring combined promoter P*_LapL_*	This study
pShuttle-LapM	pShuttleF -harbouring combined promoter P*_LapM_*	This study
pLus-Hyb	Expression vector based on P*_LapS_*	This study
pLus-His	Expression vector for expression purification	This study
pLu-bga	pLus-Hyb harbouring *bgaB*	This study
pLuHis-bga	pLus-His harbouring *bgaB*	This study
pLu-bioI	pLus-Hyb harbouring *bioI*	This study
pLuHis-bioI	pLus-His harbouring *bioI*	This study

### PCR and DNA manipulation

Polymerase chain reaction (PCR) primers and oligonucleotides used in this study are listed in Table 2. Isolation and manipulation of recombinant DNA was performed using standard techniques [24]. All enzymes were from Promega China (Beijing, China) and used as recommended by the manufacturer. The transformation of *E. coli* and *B. subtilis* was performed by electroporation [24], [25].

**Table 2:** Primers and oligonucleotides used in this study.

Primers	Sequences (5′→3′)	Restriction sites
*bgaB*-1	GCGGATCCATGAATGTGTTATCCTC	BamH I
*bgaB*-2	TTGAGCTCCTCTA AACCTTCCCGG	Sac I
spec-1	TTGGATCCGAATGGCGATTTTC	BamH I
spec-2	TTGTCGACTTGAAAAAAGTGTTTCCAC	Sal I
P43-1	TTGGGCCCTCAGCATTATTGAGTG	Apa I
P43-2	TTGGATCCCATGTGTACATTCCTCTC	BamH I
insert-1	TTGGATCCCACTTTATGGACGCC	BamH I
insert-2	TTGGGCCCATTCGGATCGTCAC	Apa I
luxS-up	TTGGATCCACTCTCCCCTTTTTTAAAAATG	BamH I
luxS-down	TTGGGCCCTAATGTTTTTGTTTCTTTTC	Apa I
luxP-up	TTGGATCCATCAAGTTCAAAGCTTTC	BamH I
luxP-down	TTGGGCCCAAAAGTAAGTTATTTTTCC	Apa I
luxM-up	TTGGATCCCACTCCTTTTTTCTTCTTTC	BamH I
luxM-down	TTGGGCCCGTTTTACAGTCAAATAAG	Apa I
luxL-down	TTGGGCCCTTATTATAATATAAGC	Apa I
*bgaB*-fus	GGCGGGATCCTACGTAGGTACC	BamH I
insert-3	TTGGATCCAGGTCAATGCTTTTC	BamH I
lux-2	TTGGATCCTCTCTCCCCTCTAATCG	BamH I
apr-1	TTGGGCCCTCAGGAGCATTTAAC	Apa I
apr-2	CTCTATTTAGGTATATCATCTCTC	
apr-3	TTGGGCCCTAAGAAAAATAGCATAC	Apa I
apr-4	CTGTATATTCAGTTTAAGGGAAG	
apr-5	TTGGGCCCTGCCAGGTTGAAG	Apa I
apr-6	AAATAGAAGGATAATATAATCTATTCC	
apr-7	TTGGGCCCAAAAATGGAAACAAAC	Apa I
apr-8	GTAGACCCAATTTTTTTCAG	
insert1-up	TAGCGAGAGATGATACAAGAACGTCCTGATCTT	
insert2-up	TACTCTTATTTGCCTCAAGAACGTCCTGATCTT	
insert3-up	TATAATAAATTAACACAAGAACGTCCTGATCTT	
insert4-up	TAAAACAATCTGAAACAAGAACGTCCTGATCTT	
apr1-down	ATCAGGACGTTCTTGTATCATCTCTCGCTATTTC	
apr3-down	ATCAGGACGTTCTTGAGGCAAATAAGAGTAGACC	
apr5-down	ATCAGGACGTTCTTGTGTTAATTTATTATAGAA	
apr7-down	ATCAGGACGTTCTTGTTTTCAGATTGTTTTATTTC	
laps-down	TTGAATTCCTCTCTCCCCTCTAATCG	EcoR I
Tag-1	CTAGACATCATCATCATCATCACAGCTAAATAAGAGCT	
Tag-2	CTTATTTAGCTGTGATGATGATGATGATGT	
bgaB-3	GCGGAATTCATGAATGTGTTATCCTC	EcoR I
bgaB-T	TTGGATCCAACCTTCCCGGCTTCATC	BamH I
bioI-1	TTGAATTCGTGACAATTGCATCGTC	EcoR I
bioI-2	TTGGATCCCACATTCTTAGGCTTATTC	BamH I
bioI-3	TTTCTAGATTCAAAAGTCACCGGCAG	Xba I

### Construction of a promoter trap vector

Using the primer pair *bgaB*-1 and *bgaB*-2, the *bgaB* coding region was PCR amplified from the plasmid pDL [6]. The amplified fragment was flanked by BamH I and Sac I at 5′ and 3′ ends, respectively. The promoter trap vector was assembled as follows. Firstly, the obtained *bgaB* digested by BamH I and Sac I was cloned into corresponding sites of pGJ103, yielding pGJ-*Bga*. Then, the Apa I-BamH I-treated spectinomycin resistance gene, which was PCR amplified from the pDG1728 [26] using the primer pair spec-1 and spec-2, was inserted into corresponding sites of pGJ-*Bga*, resulting in a promoter trap vector pShuttleF.

### Cloning of promoter fragments

The genomic DNA of *B. licheniformis* was partially digested with Sau3A I, and then ligated with the promoter trap vector pShuttleF treated with the BamH I and alkaline phosphatase. The ligation mixture was transformed into *E. coli* DH5α and recombinants harbouring promoter fragments were screened on the LB solid medium supplemented with X-Gal (20 µg/mL).

### Sequencing and analysis

Promoters were sequenced by AuGCT Biotechnology (Beijing, China). The sequence analysis was performed online with NCBI blast 2.0 (www.ebi.ac.uk); promoter region was predicted by the BPROM program (Softberry Inc., Mount Kisco, NY, USA; http://linux1.softberry.com).

### Sub-cloning of promoter regions

Using the primer pair P43-1/P43-2, the P43 promoter was PCR amplified from plasmid pB43. The resultant promoter fragment was digested with Apa I and BamH I, and cloned into corresponding sites of pShuttleF, resulting in pShuttle-P43. By means of primer pairs luxS-up/luxS-down, luxP-up/luxP-down and luxM-up/luxM-down, three *luxS* promoter fragments were cloned from genomic DNA of *B. subtilis* 1A747, *B. pumilus* BYG and *B. megaterium* W-5, respectively. The corresponding amplicons were then cloned into pShuttleF digested with Apa I and BamH I, generating pShuttle-luxS, pShuttle-luxP and pShuttle-luxM. Using insert-1/luxL-down as primers, the P*_luxS_*-i was amplified from pShuttle-09 and cloned into pShuttleF, resulting in pShuttle-luxi. The *bgaB* contained Shine–Dalgarno box (SD) was PCR amplified from pDL by using *bgaB*-fus and *bgaB*-2 and cloned into pshuttleF, resulting in pDET-1. Then, the hybrid promoter amplified from pShuttle-09 (using the primer pair insert-3/insert-2) was cloned into pDET-1 treated with Apa I and BamH I, resulting in pShuttle-Hyb. By using the primer pair lux-2/insert-2, an isolated promoter fragment (deletion of 5′ coding region of *luxS*) was amplified from pShuttle-09 and cloned into pShuttleF, yielding pShuttle-Fus1.

### Reconstruction of cloned promoter

The reconstruction of promoter was carried out by the splicing by overlapping extension PCR. Four pairs of primers, apr-1/apr-2, apr-3/apr-4, apr-5/apr-6 and apr-7/apr-8, were employed to PCR amplified −35 region of promoter P*_apr_* (promoter for the alkaline protease gene) from genomic DNA of *B. subtilis*, *B. pumilus*, *B. licheniformis* and *B. amyloliquefaciens*, respectively, yielding P*_aprS_*-1, P*_aprP_*-1, P*_aprL_*-1 and P*_aprA_*-1. Four pairs of primers (insert1-up/Lux-2, insert2-up/Lux-2, insert3-up/Lux-2 and insert4-up/Lux-2) were used to amplify the P*_luxS_* and −10 region of the hybrid promoter from pShuttle-09. The upstream primers were designed about 15 bp sequence homogenous with 3′ end of P*_aprS_*-1, P*_aprP_*-1, P*_aprL_*-1 and P*_aprA_*-1, respectively, resulting in fragments luxF-1 to luxF-4. Other four pairs of primers (apr-1/apr1-down, apr-3/apr3-down, apr-5/apr5-down and apr-7/apr7-down) were employed to amplify promoters from PaprS-1, PaprP-1, PaprL-1 and PaprA-1respectively. The downstream primers were designed about 15 bp sequence homogenous with 5′end of −10 region of the hybrid promoter, resulting fragment PaprS-2, PaprP-2, PaprL-2 and PaprA-2. With the mixture pairs of P*_aprS_*-2/luxF-2, P*_aprP_*-2/luxF-3, P*_aprL_*-2/luxF-4 and P*_aprA_*-2/luxF-5 as templates, in which there were about 30 bp overlap in each mixture pair, four overlapping PCR amplifications were facilitated by means of apr-1/Lux-2, apr-3/Lux-2, apr-5/Lux-2 and apr-7/Lux-2, respectively. Recombined promoters P*_LapS_*, P*_LapP_*, P*_LapL_* and P*_LapA_* were digested with Apa I and BamH I and cloned into pShuttleF, respectively, resulting in pShuttle-P*_LapS_*, pShuttle-P*_LapP_*, pShuttle-P*_LapL_* and pShuttle-P*_LapA_*.

### Construction of expression vectors

Using primers apr-1 and laps-down, the promoter P*_LapS_* flanked by Apa I and EcoR I was PCR amplified from pShuttle-P*_LapS_*. The resultant promoter was digested with Apa I and EcoR I and cloned into corresponding sites of pGJ103, yielding the expression vector pLus-Hyb. To introduce 6-His tag, synthetic oligonucleotides Tag-1 and Tag-2 were annealed. The annealed fragment with adhesive ends of Xba I and Sac I at 5′ and 3′ ends respectively was inserted into the Xba I-Sac I-treated pLus-Hyb, resulting in pLus-His.

Using primer pairs bgaB-3/bgaB-2 and bgaB-3/bgaB-T, two *bgaB* fragments were PCR amplified from pShuttleF, resulting in bgaF-1 flanked with EcoR I and Sac I and bgaF-2 with EcoR I and BamH I. bgaF-2 did not contain the termination codon. The bgaF-1 and bgaF-2 were cloned into pLus-Hyb and pLus-His, respectively, yielding pLu-*bga* and pLuHis-*bga*. Similarly, the *bioI* gene were PCR amplified from *B. subtilis* 1A747 genomic DNA by using primer pairs bioI-1/bioI-2 and bioI-1/bioI-3, resulting in bioIF-1 with EcoR I and BamH I and bioIF-2 with EcoR I and Xba I. The resultant fragments were inserted into pLus-Hyb and pLus-His, yielding pLu-*bioI* and pLuHis-*bioI*.

### β-Gal activity assay

The β-Gal activity assay was carried out as previously described [27]. Samples were taken at different time points of culture and the β-Gal activity was measured. The activity was expressed as Miller units per mL sample (Miller U/mL). For each assay, three independent experiments were performed with two replicates. Statistical tests were carried out by the SPSS (Statistical Product and Service Solutions) software. Data are presented as means± S.D.

## Results

### Screening and isolation of the strong promoter fragments from *B. licheniformis* chromosomal DNA

**Figure 1:**
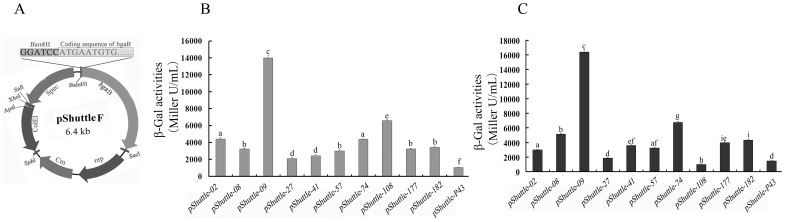
A map of the promoter trap vector pShuttleF (A) and β-Gal activities of 10 selective positive clones in *E. coli* (B) and *B. subtilis* (C). (A) The rep represents the replication protein in *B. subtilis*. ColEI, *bgaB* and Cm represent the *E. coli* ColEI replicon, the coding sequence of β-Gal and chloramphenicol-resistance marker, respectively. The unique restriction sites are marked on the outside of the map. (B). Production of β-Gal from 10 selective positive clones in *E. coli* DH5α after 24 h culture. Different letters on columns indicate significant differences (p<0.05). (C). Production of β-Gal from 10 selective positive clones in *B. subtilis* 1A747 after 24 h culture. Different letters on columns indicate significant differences (p<0.05).

To screen and isolate strong promoter elements, a promoter trap vector pShuttleF (Figure 1A) was constructed on an *E. coli-B. subtilis* shuttle vector pGJ103. The pShuttleF contained *bgaB* coding for a heat stable β-galactosidase (β-Gal) as a reporter gene. In addition, an alternative resistance selection marker, spectinomycin, was introduced into the pShuttleF beside the chloramphenicol resistance marker. Both spectinomycin and chloramphenicol resistance genes could confer the resistance selection either in *E. coli* or *B. subtilis*. Finally, an engineered BamH I restriction site was designed next to the start codon of *bgaB* in order to clone DNA fragments digested by the Sau3A I. Validation of the pShuttleF was achieved by inserting a commonly used strong promoter P43 (2, 6) in *B. subtilis* upstream of *bgaB*. The resultant pShuttle-P43 was transformed into *E. coli* DH5α and *B. subtilis* 1A747 and blue colonies on solid LB plate supplemented with X-gal were isolated. The *bgaB* directed by the P43 in pShuttleF was duly expressed in *E. coli* and *B. subtilis* (Figures 1B and 1C).

In order to identify new promoters, Sau3A I-digested *B. licheniformis* DNA fragments were inserted into the BamH I site of the promoter trap vector pShuttleF and resultant plasmids were electro-transformed into *E. coli* DH5α. Two hundred colonies were picked from about 1000 colonies according to visible coloration on the X-gal screen plate in the presence of both antibiotics. The β-Gal activities by these recombinants after 24 h of culture ranged from 500 Miller U/mL to 14016 Miller U/mL. Amongst them, the recombinant pShuttle-09 demonstrated the highest β-Gal production. The selected 200 colonies were also electro-transformed into the *B. subtilis*1A747. Ten blue colonies were selected to measure the β-Gal activities. As shown in Figure 1C, pShuttle-09 again showed the highest β-Gal activity with the activity of 16417±300 Miller U/mL at 24 h, which was eight times of that from the P43 promoter system in *B. subtilis*. To further verify the reporter gene product driven by pShuttle-09, SDS-PAGE analysis of the β-Gal from the pShuttle-09 both in *E. coli* and *B. subtilis* was carried out. Coomassie blue staining revealed a distinct band with a molecular mass of approximately 70 kDa, which corresponded to the molecular weight of β-Gal (data not shown). Taken together, these results suggested that pShuttle-09 contained a putative strong promoter from *B. licheniformis* chromosomal DNA and drove expression of the reporter gene both in *E. coli* and *B. subtilis*.

### Sequence analysis, predication and characterization of the cloned promoter fragment

**Figure 2:**
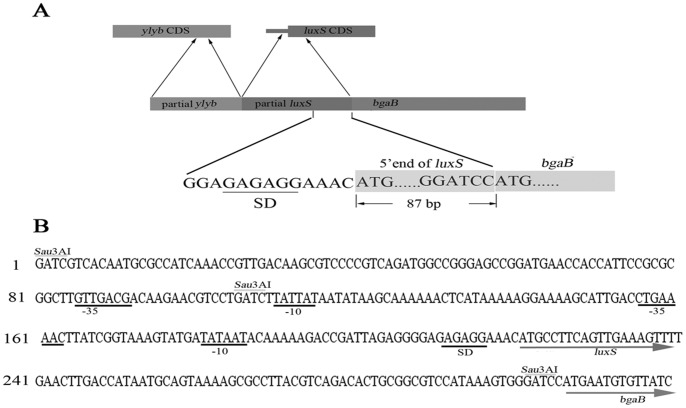
A map (A) and the sequence (B) of an inserted fragment in pShuttle-09. (A) *ylyb* CDS is the coding sequence of *ylyb*; *luxS* CDS represents the coding sequence of *luxS*; Partial *luxS* includes 5′ non-translated region and an 87 bp coding sequence of *luxS*. (B) The conservative regions of two putative promoter (−10 and −35) are underlined. The Sau3AI restriction sites are indicated. The beginnings of open reading frames of *luxS* and *bgaB* under the putative promoters are indicated by arrows.

To characterize the putative promoter DNA element in pShuttle-09, the inserted fragment was enzyme cut and sequenced. The sequencing result showed that the inserted fragment was 305 bp long. Sequence alignment showed that the inserted sequence in pShuttle-09 belonged to *B. licheniformis* genome sequence (NC_CP000002.3) with 96% of homology. According to the annotation of *B. licheniformis* genomic DNA sequences, the inserted sequence contained two fragments (Figure 2A) corresponding to a partial sequence of the coding region of *ylyB* gene (about 109 bp) and a 5′end partial sequences of *luxS* (about 200 bp).

Sequence analyses (Figure 2B) also showed truncated *luxS* gene was fused with *bgaB* and a typical SD was present 7 bp upstream of the start codon of the *luxS* gene. It contained typical −35 and −10 elements recognized by a Sigma Factor σ^A^. Therefore, the promoter P*_luxS_* might be the control element of *bgaB* in pShuttle-09. However, further in silico prediction of promoters (Figure 2B) indicated two conserved prokaryotic promoter regions with high probability scores in the cloned fragment. The first putative promoter (P*_luxS_*) was located in the 5′ non-translational region of *luxS* gene. Interestingly, the second putative promoter was located on two sides of the second Sau3A I-cutting site in pShuttle-09. The −35 element was located within the coding region of *ylyB*, while the −10 element was located upstream of the core promoter region of *luxS*.

**Figure 3:**
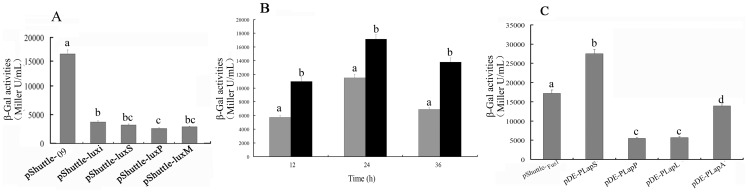
β-Gal activities from P*_luxS_* (A), the hybrid promoter (B) and the reconstructed promoters (C). (A) Production of β-Gal from pShuttle-09, pShuttle-luxi, pShuttle-luxS, pShuttle-luxP and pShuttle-luxM in *B. subtilis* 1A747 after 24 h culture. Different letters on columns indicate significant differences (p<0.05). (B) Production of β-Gal from pShuttle-Hyb (gray columns) and pShuttle-Fus1 (black columns) in *B. subtilis* 1A747 after different hours of culture. Different letters on columns in the same time point indicate significant differences (p<0.05). (C) Production of β-Gal from pShuttle-Fus1, pDE-P*_LapS_*, pDE-P*_LapP_*, pDE-P*_LapL_* and pDE-P*_LapA_* in *B. subtilis* 1A747 after 24 h culture. Different letters on columns indicate significant differences (p<0.05).

In order to determine the effect of the first putative promoter (P*_luxS_*) on the reporter gene expression, a P*_luxS_*-i vector was constructed by sub-cloning a 200 bp P*_luxS_* fragment into pShuttleF. For facilitating a rigorous comparison, promoters of *luxS* were also cloned from genomic DNA of *B. subtilis*, *B. pumilus* and *B. megaterium*. The β-Gal production driven by P*_luxS_* from *B. subtilis*, *B. pumilus* and *B. megaterium* were about 78%, 63% and 70% of that from *B. licheniformis* (Figure 3A), respectively. However, the β-Gal production driven by P*_luxS_* from *B. licheniformis* was only about 27% of that from pShuttle-09 in *B. subtilis* (Figure 3A). These data strongly suggested that P*_luxS_* is not a major promoter for β-Gal expression in pShuttle-09.

Analysis of the second putative promoter showed the −10 region was located at 6 bp in the second Sau3A I-cutting region and −35 region was located at 86 bp in the first Sau3A I-cutting region in pShuttle-09. A partial sequence of the coding sequence of the *ylyB* gene by itself could not have the promoter activity, since it did not contain complete promoter elements. It appears that the hybrid promoter was unintentionally created in pShuttle-09. Subsequently, this hybrid promoter was sub-cloned to determine whether it can direct the expression of β-Gal or not. As shown in Figure 3B, the β-Gal production driven by the hybrid promoter was about 67% of that from pShuttle-09 in *B. subtilis*. It seems that both the hybrid promoter and P*_luxS_* contributed additively to expression of β-Gal in pShuttle-09.

### Improvement of the cloned double promoter through reconstruction of the hybrid promoter element

In order to further improve efficacy of the double promoter, the second Sau3A I-cutting region containing P*_luxS_* and another −10 region was assembled with −35 regions of P*_Apr_* from *B. subtilis*, *B. pumilus*, *B. licheniformis* and *B. amyloliquefaciens*, respectively. The corresponding promoters, P*_LapS_*, P*_LapP_*, P*_LapL_* and P*_LapA_*, were used to drive the expression of β-Gal. The results (Figure 3C) indicated that β-Gal activity driven by P*_LapS_* was 1.6 fold higher than that from pShuttle-09 after 24 h culture. On the other hand, the production of β-Gal from P*_LapA_*, P*_LapP_* and P*_LapL_* was only about 81%, 32% and 33% of that from pShuttle-09, respectively. Subsequently, P*_LapS_* was used for construction of expression vectors.

### Construction of expression vector using the improved promoter P*_LapS_*

**Figure 4:**
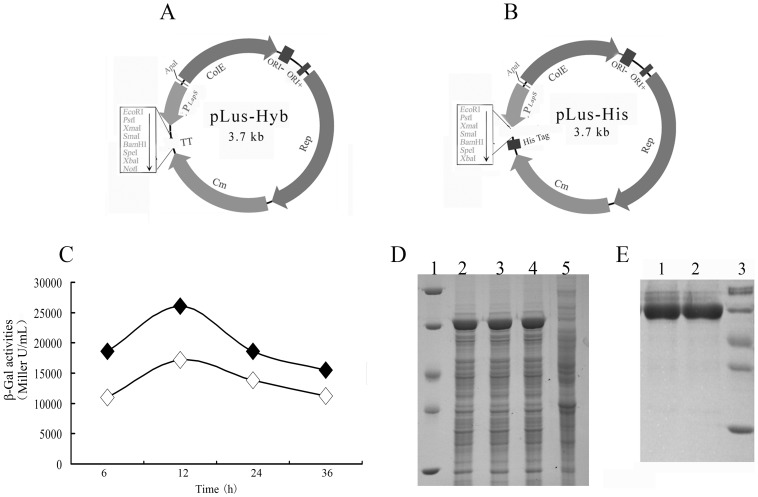
Maps of expression vectors pLus-Hyb (A), pLus-His (B), the β-Gal activity from the pLu-bga (C), SDS-PAGE analyses of BgaB in crude extract (D) or after purification (E). (A) ORI+, ORI− and rep represent the single-strand replication origin, the double strand origin and the replication protein in *B. subtilis*, respectively. ColEI and Cm represent the *E. coli* ColEI replicon and a chloramphenicol-resistance marker. The unique restriction sites are marked on the outside of the map. P*_LapS_* represent the improved promoter. (B) His Tag represents the 6 histidine tag. (C) Production of β-Gal from pShuttle-Fus1 (◊) and pLu-bga (♦) in *B. subtilis* 1A747, respectively. (D) Lane 1, molecular mass markers (top to bottom: 116, 66, 45, 35 and 25 kDa); Lanes 2, 3 and 4, crude extract of three replicate cultures of *B. subtilis* 1A747 harbouring pLu-bga harvested at 24 h; lane 5, crude extract of *B. subtilis* 1A747 harbouring pLus-Hyb as a negative control. (E) Lanes 1 and 2, purified β-Gal from *B. subtilis 1A747* harbouring pLuHis-bga; Lane 3, molecular mass markers (top to bottom: 116, 66, 45, 35 and 25 kDa).

To further exploit its application in *B. subtilis*, the strong promoter P*_LapS_* was sub-cloned as a truncated fragment flanked with engineered Apa I and EcoR I restriction sites, with the EcoR I site close to the SD. The resultant promoter was assembled to *E. coli*-*B. subtilis* shuttle vector pGJ103, generating an expression vector pLus-Hyb (Figure 4A). In order to easily detect and purify expressed protein, another vector pLus-His (Figure 4B) was constructed through introducing 6-his tag at the end of MCS in pLus-Hyb.

**Figure 5:**
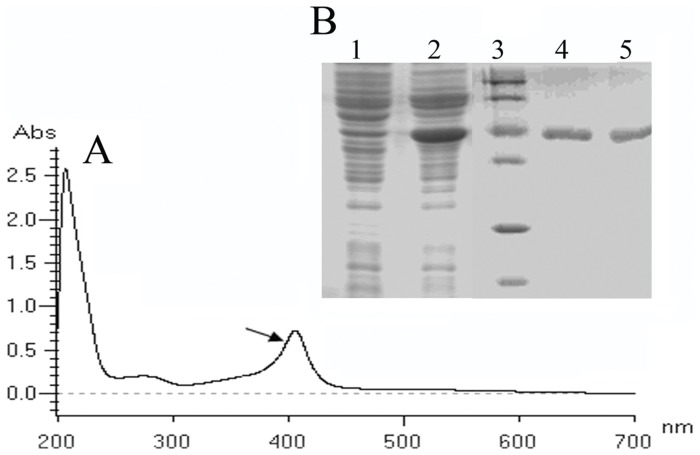
Wavelength scan analysis of Biol production from *B. subtilis*1A747 harbouring pLu-bioI (A) and SDS-PAGE analyses of Biol production from crude extract or purified Biol of *B. subtilis*1A747 harbouring pLu-bioI and pLuHis-bioI, respectively (B). (A) The arrow indicating the characteristic absorption peak of BioI. (B) Lane 1, crude extract of *B. subtilis* 1A747 harbouring pLus-Hyb as a negative control; Lane 2, crude extract of *B. subtilis* 1A747 harbouring pLu-bioI; Lane 3, molecular mass markers (top to bottom: 116, 66, 45, 35 25 and 18 kDa); Lanes 4 and 5, purified β-Gal from *B. subtilis 1A747* harbouring pLuHis-bioI.

To determine efficiencies of pLus-Hyb and pLus-His, bgaF-1 and bgaF-2 were cloned into pLus-Hyb and pLus-His, respectively, to facilitate β-Gal expression. The production of β-Gal from the pLu-bga reached 26037±1037 Miller U/mL (Figure 4C), indicating that the β-Gal was highly expressed. SDS-PAGE analyses of β-Gal from crude extract of *B. subtilis* harbouring pLu-bga (Figure 4D) or purified β-Gal from *B. subtilis* harbouring pLuHis-bga (Figure 4E) further verified that β-Gal was successfully expressed. Similarly, *bioI* gene involved in biotin biosynthesis pathway of *B. subtilis* was successfully expressed by using two similar expression vectors (pLu-biol and pLuHis-biol) in *B. subtilis* (Figure 5). Therefore, these results further demonstrated the effectiveness of the promoter systems for protein expression.

## Discussion

The major finding from this study is that an artificial double promoter was obtained from *B. licheniformis* through a promoter trap system. The double promoter contained P*_luxS_* and a hybrid promoter and can be used for protein expression in *B. subtilis*. These results support the notion that it is possible to shuffle, generate and select a strong prokaryotic promoter simultaneously, by applying a promoter trap system to randomly cut DNA fragments from a genome.

The interest in promoters stems from myriad opportunities for controlling gene expression. Our promoter trap system was designed to increase the stringency of screening by employing the coding region of *bgaB* rather than the *bgaB* gene with a SD as a reporter. So far, the promoter-less *bgaB* gene has been used as a reporter in several promoter trap systems [11], [22]. However, others usually used *bgaB* gene with a SD. In our system, promoter fragments inserted into the upstream of *bgaB* could not drive expression of β-Gal unless the cloned promoter fragments contain SD. This design might limit the number of recombinant clones which can be screened. Nevertheless, approximately 1000 blue recombinant colonies were observed in this study. The strength of cloned promoters was conveniently determined via coloration caused by production of β-Gal when a recombinant harbouring a promoter fragment was cultured on solid LB supplemented with X-gal. Further quantitative determination of putative promoter strength was easily achieved by measuring the activity of β-Gal.

*B. licheniformis* genomic DNA was used as a potential promoter source in this study. Recombinant protein expression in *B. subtilis* can be driven by promoters from *B. subtilis* or from other bacillus species. To date, several strong exogenous promoters for the *B. subtilis* system have been isolated and used to construct expression vectors [3], [12], [28]. For example, exogenous promoters P*_xyl_* and P*_spac_* have been widely used as expression control elements in *B. subtilis* [12], [28]. Our results support the notion that other bacillus species could be good sources of strong promoters for *B. subtilis*.

Our results exemplified the potential of a promoter trap system to create a strong hybrid promoter. A hybrid promoter is defined in this study as a promoter consisting of different core elements from two different genes. One promoter identified in this study is a hybrid promoter assembled artificially from a −35 region from the coding sequence of *ylyB* gene and a −10 element from non-coding sequence of *luxS* gene. Our hybrid promoter is artificially created and quite different from reported hybrid promoters, which contain elements from two different promoters. It is well known that a hybrid promoter could be far more efficient than either one of the parental promoters [29]. Recently, Kim [30] constructed a hybrid promoter, BJ27UP, from a strong promoter BJ27D88 and a fragment of the *tac* promoter in order to express *B. licheniformis* aminopeptidase in *B. subtilis* and found that the activity of the hybrid promoter was increased approximately threefold in comparison with the *tac* promoter. Nevertheless, the study on hybrid promoters is limited, presumably due to lack of suitable ways to create hybrid promoters.

The results from this study also demonstrated the capability of a promoter trap system to create a double promoter. Interestingly, both the hybrid promoter and P*_luxS_* promoter in our study contributed to transcription of the reporter gene in an additive way. A double promoter in this study is defined as the combination of two promoters which control transcription of same gene. This definition is different from most overlapping promoters often seen in bacteria. Analyzing the database of the *E. coli* genome, Bendtsen [31] found 14% of the identified ‘forward’ promoters overlap with a promoter oriented in the opposite direction. These overlapping promoters are arranged in three different ways: (i) each promoter transcribes a gene and one or more regulatory proteins are identified which affect transcription of at least one of the promoters directly; (ii) each promoter transcribes a gene but no regulatory proteins are known to bind the promoter region; and (iii) only one of the promoters transcribe an annotated gene, while the other promoter could transcribe a regulatory antisense RNA or act as a regulatory RNAP binding site which interferes with the promoter transcribing the annotated gene [31]. However, some overlapping promoters do control same gene. For example, Wang and Doi [32] detected two overlapping promoters for the chloramphenicol acetyltransferase gene and these two promoters were recognized by two different RNA polymerase holoenzymes. The authors further proposed that genes expressed during both growth and sporulation may be regulated by tandem overlapping promoters whose transcription initiation points are either identical or very close. The possible benefit of double promoters to increase transcription has been explored in biotechnological application. Most studies have showed that two or more tandem promoters could significantly improve the expression level of heterogeneous genes. Widner [33] found that gene expression was distinctly increased under the control of two or three tandem promoters in contrast to one alone in *B. subtilis*. Kang [34] also reported that two expression systems constructed by sequential alignment of a constitutive promoter for either α-amylase from *B. subtilis* NA64 or maltogenic amylase from *B. licheniformis* downstream of the Hpa II promoter elevated the TSaGT productivity by 11- and 12-fold, respectively, in comparison with the single Hpa II promoter system. Li [35] tried to construct multiple core-tac-promoters (MCPtacs) in tandem and found that integration of the *phaCAB* genes with the 5 copies of core-tac-promoters resulted in an engineered *E. coli* that can accumulate 23.7% polyhydroxybutyrate of the cell dry weight in batch cultivation. On the other hand, Wu [36] did not observe improvement of expression, when two tandem-linked promoters were used. Our results support the idea to use double promoters to increase production efficiency of recombinant proteins. Whether the double promoter in this study is targeted by same RNA polymerase or different RNA polymerases is not known at this time and worthy of further investigation.

In conclusion, our data support the concept of using a promoter trap system to discover new promoters. The usefulness of a promoter trap system is likely due to random shuffling and recombination of DNA fragments and adoption of a rapid and high-throughput screening. It is tempting to speculate that more novel promoters could be found if reaction conditions for cutting and ligation could be adjusted to increase chances for shuffling DNA fragments.
